# Refining the Estimate of Fruit Consumption in Brazil’s National Food Consumption Survey: Quantifying the “Cherry on the Cake”

**DOI:** 10.1016/j.cdnut.2026.109413

**Published:** 2026-06-25

**Authors:** Thaís R Guastalle, Glenda V da Silva, Mônica CR dos Anjos, Claudia CB Almeida, Victoria P de Quadros, Agnieszka Balcerzak, Bridget A Holmes, Sandra P Crispim

**Affiliations:** 1 Postgraduate Program in Food and Nutrition, Federal University of Paraná, Curitiba, Paraná, Brazil; 2 Food and Nutrition Division, Food and Agriculture Organization of the United Nations (FAO), Rome, Italy

**Keywords:** fruit, recipe, food ingredient, food consumption, 24-hour dietary recall, dietary survey

## Abstract

**Background:**

Cultural differences can influence how foods are defined, classified, and quantified, making it challenging to compare and interpret dietary data on national and global levels. Data harmonization plays a crucial role in addressing these challenges, and the FAO/WHO GIFT (Global Individual Food Consumption Data Tool) platform provides a framework for promoting harmonized data sharing.

**Objectives:**

This observational study aimed to quantify fruit consumption in Brazil in a harmonized manner, supporting national assessments and contributing to global dietary data-sharing efforts.

**Methods:**

We analyzed 24-hour dietary recall data from the latest Brazilian National Food Consumption Survey (Inquérito Nacional de Alimentação/Pesquisa de Orçamentos Familiares) across 3 analytical scenarios to progressively improve comparability and detail about fruit consumption trends and consumed varieties: *1*) original data, *2*) data with harmonized food group classification, and *3*) data with harmonized food group classification and recipe disaggregation.

**Results:**

In total, we identified 85 distinct fruit types. The 5 most consumed fruits in Brazil were banana (22.8%), orange (16.2%), apple (12.8%), papaya (9.1%), and açai berry (7.3%). Notably, açai berry stood out as the most consumed fruit in the North region (36.7%). Recipe disaggregation revealed 77 recipes containing fruits (7.9% of the total reported fruit consumption) and included 22 different fruits as ingredients. This significantly increased the number of identified fruit consumers in Brazil, with proportions of consumers rising from 37.9% [95% confidence interval (CI): 37.0%, 38.9%] in scenario (1) to 52.2% (95% CI: 51.2%, 53.2%) in scenario (3). The mean daily fruit consumption per capita was estimated at 79.8 g/d (95% CI: 77.3 g/d, 82.3 g/d) and 49.7 g/1000 kcal/d (95% CI: 48.2 g/1000 kcal/d, 51.3 g/1000 kcal/d).

**Conclusions:**

The analysis reveals substantial variations in fruit consumption across regions, sex, age groups, education levels, income levels, and ethnic groups. This study highlights the critical importance of food group classification and recipe disaggregation in measuring fruit consumption, thereby increasing the comparability of data within and between countries through data harmonization.

## Introduction

Accurate data on food consumption play a vital role in understanding dietary intakes and patterns, assessing nutritional indicators, and formulating effective public policies. However, challenges emerge in the collection and use of these data because of the diverse ways in which foods are defined and classified [1,2], which can be particularly influenced by cultural nuances. For instance, the classification of specific foods, such as plantains and olives, can differ between dietary guidelines and botanical classifications. In regions with high plantain consumption, like Central America, the Caribbean, the Pacific Islands, and some African countries [3,4], plantains are commonly classified as “roots and tubers” in dietary guidelines. This contrasts with their botanical classification as fruits, as considered in the Brazilian National Food Consumption Survey (INA) [5]. The same applies to olives, which are classified as fruits in some contexts but not in others [5,6]. These inconsistencies illustrate how such differences can be challenging for data comparison and interpretation at the international level.

Fruit consumption is a key dietary indicator, widely used to assess diet quality and associated with reduced risk of noncommunicable diseases, making it particularly relevant for global monitoring and nutrition policy development. Moreover, fruit consumption has been increasingly recognized within a broader food systems perspective, where production methods, processing, accessibility, and dietary patterns influence both human and planetary health [7,8].

Given the potential variability in fruit classification, it is noteworthy that data on fruit consumption are well captured in dietary surveys. In fact, a study by Miller et al. [9] covering 1220 surveys showed that over 96.2% of the analyzed surveys captured fruit intake. However, in several countries, fruit and vegetable consumption is measured together, typically in terms of portions, frequency, or weight [[10], [11], [12]]. A study by Frank et al. [13] involving 28 low- and middle-income countries (LMICs) found that 32% of the analyzed surveys collected data on fruit and vegetable consumption together, without reporting them separately. These studies also revealed variations in data collection methods, including food frequency questionnaires (FFQs), 24-h dietary recalls (24HR), and dietary records, which can lead to differing definitions and food group classifications. This complexity further complicates comparisons between studies and influences consumption recommendations [1], underscoring the significance of assessing fruit consumption separately from vegetable consumption.

Another relevant challenge concerns data comparability across food classification systems. For example, findings from the Italian National Food Consumption Survey using the FoodEx2 food classification and description system [14] demonstrated that 67 fruit codes from the original survey data were consolidated into 55 unique FoodEx2 codes. The identification of synonymous terms played a key role in enhancing comparability at both national and international levels [15].

A further source of complexity lies in how recipes are reported in dietary surveys. Recipes, either prepared at home or commercially, consist of multiple ingredients that can be separated, classified into different food groups, and quantified [16]. The way fruits are consumed, whether on their own or as part of a dish, impacts dietary assessment. In this context, failing to identify fruits consumed as part of recipes may result in an underestimation of fruit consumption in surveys [12,17,18].

In response to these challenges, the Food and Agriculture Organization of the United Nations (FAO) and the World Health Organization (WHO) have taken significant steps to introduce parameters for the harmonization of dietary survey data. This harmonization was necessary to disseminate existing dietary survey data through the FAO/WHO GIFT platform, which stands for the Global Individual Food Consumption Data Tool [19]. This platform serves as a global repository for individual-level quantified food consumption data, enabling experts to share and access information, thereby supporting national and global monitoring and policy formulation [20]. The platform incorporates a specific food classification system [14] designed to harmonize data and uses standardized food groupings in data analysis, aiding meaningful global analyses and comparisons [19,21]. FAO/WHO GIFT’s applicability has been demonstrated in recent work using harmonized dietary data for international comparisons [[22], [23], [24], [25]] and has been further expanded through its integration with other global initiatives, such as the Global Dietary Database [26]. Furthermore, disaggregation of recipes into their individual ingredients is encouraged for accuracy in dietary assessments, although a food group for composite dishes/recipes is also included in the food classification system used by this platform [19].

In this context, the present study utilizes data from the Brazilian National Food Consumption [Inquérito Nacional de Alimentação/Pesquisa de Orçamentos Familiares (INA/POF)] 2017–2018 survey to identify fruits consumed by the studied population. Specific objectives include assessing the impact of recipe disaggregation on the fruit food group consumption estimates, identifying synonymous and homonymous terms in fruit consumption reports, and estimating fruit consumption among different sociodemographic groups. By applying the harmonized nutrition-sensitive food grouping used by FAO/WHO GIFT, this research ultimately aims to provide insights into the patterns of fruit consumption in Brazil for different strata, contributing to more accurate dietary assessments and fostering global data harmonization.

## Methods

The study design was cross-sectional, focusing on the reanalysis of data from the INA of the Household Budget Survey (POF) for the years 2017–2018. The POF, conducted by the Ministry of Health in collaboration with the Brazilian Institute of Geography and Statistics (IBGE), took place between 11 July, 2017, and 9 July, 2018. The INA was a module for assessing individual quantitative food consumption within the POF, and the collected data follow the probabilistic sampling of the main survey, which includes a master sample and a representative subsample from the 5 macroregions of Brazil [5].

**FIGURE 1:**
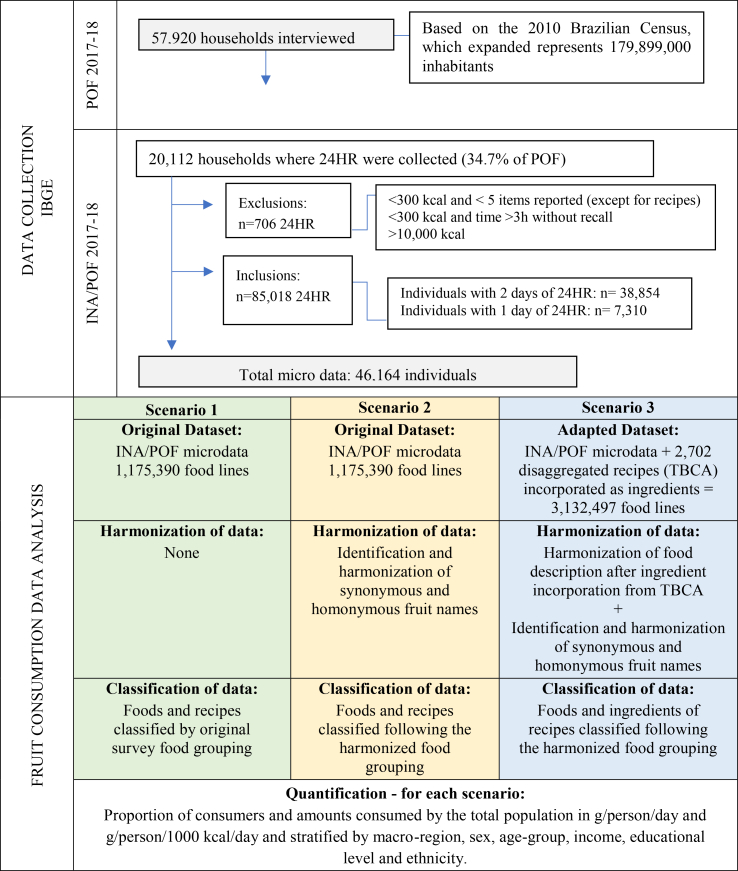
Flowchart of data collection from the Brazilian National Food Consumption Survey (INA/POF) 2017-2018 and fruit consumption data analysis. 24HR, 24-hour dietary recalls; IBGE, The Brazilian Institute of Geography and Statistics; INA, Brazilian National Food Consumption Survey; POF, Household Budget Survey; TBCA, Brazilian Food Composition Table.

Food consumption data were collected from 20,112 households, comprising a 34.7% subsample of the overall 57,920 households in the POF (Figure 1). The study included individuals aged ≥10 y, encompassing both urban and rural areas. The study excluded individuals living in institutionalized residences [5]. All participants provided informed consent prior to data collection. Because the current study used publicly available deidentified data, additional ethical approval was not required.

### Dietary assessment at INA/POF

Trained interviewers applied the multiple-pass method to collect 2 nonconsecutive 24HR. They used tablets equipped with software called BrasilNutri, incorporating 1832 food and beverage entries. BrasilNutri has demonstrated acceptable performance in estimating energy intakes [27]. The 24HR interviews collected data on the name, preparation method, and quantity of foods and beverages consumed, as well as the time and location of consumption. Interviewers also asked follow-up questions regarding the addition of ingredients such as olive oil, butter, margarine, sugar, sweetener, honey, molasses, mayonnaise, ketchup, mustard, soy sauce, grated cheese, and cream to the reported foods. Methods of quantification included household measurements, standard units, and standard portions, which were later transformed into grams of consumption [5].

Within the framework of the INA, a total of 706 24HR were excluded because of inconsistencies. All records with energy intake below 300 kcal or above 10,000 kcal were evaluated for plausibility. Records with energy intake below 300 kcal were considered incomplete and excluded if they contained <5 reported items or included periods longer than 3 h without any reported consumption. Records with energy intake above 10,000 kcal were excluded when implausible quantities or units of measurement were identified (e.g., unrealistically large portion sizes or reporting multiple cups of energy-dense foods in a single eating occasion, suggesting data-entry or coding errors) [5]. The final analytical sample comprised 46,164 participants, of whom 38,854 (84.2%) completed both days of data collection (Figure 1).

The microdata for the 24HR is available on the IBGE website (https://www.ibge.gov.br), consisting of a comprehensive database with 1,175,390 lines of reported foods from the INA/POF 2017–2018. The collected data were matched with quantities of dietary components from the Brazilian Food Composition Table (TBCA) [28]. Additionally, the TBCA team developed a database containing 2702 disaggregated recipes, which were used to quantify the final energy and nutrient amounts of the preparations and recipes reported during the INA/POF survey. Recipes are defined as composite dishes or mixed dishes, which represent the combination of ingredients from different food sources [16]. Although individual ingredients from recipes are not on the IBGE site, researchers can obtain them by contacting the data compilers and signing a data use agreement.

The energy and nutrient composition of the recipes and preparations used in INA/POF was estimated using 3 methods: direct, indirect, and mixed. The direct method estimates the composition based on known ingredients and preparation processes, whereas the indirect method uses raw ingredient data for more complex dishes, such as bread and cakes. The mixed method is applied when ingredients in a recipe undergo different cooking methods [28]. More details on these procedures are provided by Coelho et al. [29], who describe the methodology adopted in the TBCA. Briefly, standardized recipes were compiled from national recipe manuals and technical food preparation documents. Nutrient composition was estimated using the edible portion of ingredients, applying yield factors, nutrient retention factors, and fat absorption or loss factors when appropriate. When recipe-specific information was unavailable, standardized ingredient proportions and preparation procedures were used to ensure consistency and comparability across preparations.

### Data analysis

Fruit consumption was quantified using 3 scenarios. In the first scenario, we analyzed the original data from the INA/POF survey, using the original food grouping. In the second scenario, foods in the dataset were reclassified according to the harmonized nutrition-sensitive food grouping used by FAO/WHO GIFT [21], hereafter referred to as the harmonized food grouping. The third scenario involved an adapted dataset that was also reclassified using the harmonized food grouping, but with disaggregated recipes from the TBCA. This adaptation replaced each recipe entry with multiple entries, each representing its individual ingredients, resulting in a dataset of 3,132,497 lines. Thus, although the first and second datasets reflected fruit consumption without recipe disaggregation, the third dataset accounted for all ingredients that formed the recipes. Fruit consumption in all scenarios was subsequently quantified, as summarized in Figure 1.

To quantify fruit consumption in the second and third scenarios, we examined the reported food list for fruits consumed as a discrete item or as ingredients in recipes such as salads, drinks, and savory preparations. During this phase, we encountered synonymous fruit names resulting from regional conventions and species diversity. Some synonymous fruit names were merely variations in spelling (e.g., “*siriguela*” and “*seriguela*”), whereas others involved different names for the same species (e.g., “*pinha*” and “*fruta do conde*,” both referring to *Annona squamosa*). All these were harmonized to represent a single fruit entity. In addition, fruit name homonyms were investigated in the reported food list, referring to terms that share the same spelling but denote different fruits. This is particularly relevant in Brazil, where linguistic diversity may lead to ambiguity in food reporting.

We also addressed inconsistencies between the descriptions found in the TBCA ingredient list and those used in the INA/POF. For instance, the TBCA’s detailed description “*apple, with peel, in natura*” was simplified in the INA/POF to “*Apple*.” In other cases, the composition of ingredients in the TBCA differed from the INA/POF, likely because of a lack of exact matches, leading to the use of similar food items for linkage. For example, “pequi” (*Caryocar brasiliense*) was linked to “piquiá” (*Caryocar villosum*).

After identifying and harmonizing fruit descriptions in scenarios 2 and 3, all fruits, whether consumed individually or as ingredients, were classified according to the harmonized food grouping, under the group “Fruits and their products,” which is listed as group 10 among 19 possible groups [21]. This group is further divided into 5 subgroups (Table 1): *1*) yellow- and orange-fleshed fruits (excluding citrus), *2*) citrus fruits, *3*) other, mixed, and unspecified fruits, *4*) processed fruits (excluding dried and candied), and *5*) dried fruits (excluding candied).

**TABLE 1:** FAO/WHO GIFT nutrition-sensitive classification of fruit group and subgroups

10. Fruits and their products	Description	Examples

1001. Yellow- and orange-fleshed fruits (excluding citrus): fresh	Yellow- and orange-fleshed fruits, excluding citrus fruits; fresh and after cooking, but not after drying, pickling, candying, or other preservation methods.	Mango, papaya, and passion fruit
1002. Citrus fruits: fresh	Citrus fruits; fresh and after cooking, but not after drying, pickling, candying, or other preservation methods.	Orange, lemon, lime, tangerine, and grapefruit
1003. Fruit—other, mixed, and unspecified	Fruits, excluding yellow- and orange-fleshed fruits, citrus, and starchy fruits, as well as fruits of mixed and/or unspecified origin. This group includes fresh and cooked fruits, but not fruits that have been dried, pickled, candied, or preserved using other methods.	Apple, banana, watermelon, grape, and açai berry
1004. Fruits—all types: processed (excluding dried and candied)	Processed (excluding dried, candied, and fermented) yellow- and orange-fleshed fruits, citrus and other fruits, excluding starchy fruits, but including canned, jarred and pickled fruits, and fruits preserved in vinegar brine.	Canned fruits, fruit in syrup, and olives
1005. Fruits—all types: dried (excluding candied)	Dried yellow- and orange-fleshed fruits, citrus, and other fruits (excluding starchy fruits), but not candied fruits.^1^	Raisins, dried apricots, and dried prunes

Abbreviation: FAO/WHO GIFT, Global Individual Food Consumption Data Tool.

1 Candied fruit: considered in the Sweets and sugars group.

According to the above food grouping, processed fruits with added sugar (e.g., jams, marmalades, and chocolate-coated fruits) are classified as sweets rather than fruits. In addition, fruit juices, nectars, and fruit-based beverages (e.g., smoothies) are classified as beverages. Thus, in line with the harmonized food grouping, this study excludes recipes containing processed fruits with added sugar, as well as fruit juices and fruit-based beverages. These items were also not included in scenario 1, which was based on the original INA/POF classification. Furthermore, all fruit descriptions were harmonized using the FoodEx2 system [14], which facilitated application of the harmonized food grouping, as FoodEx2 food names are standardized and described in English using common terminology, and the codes are mapped to the harmonized food grouping, enabling automatic classification.

Fruits originating from sociobiodiversity were further identified in the third scenario, following the Brazilian Interministerial Ordinance, Ministry of Agriculture, Livestock and Food Supply/Ministry of the Environment and Climate Change n. 10/2021 [30]. This ordinance lists 92 native species of sociobiodiversity acknowledged for their nutritional value and suitability for commercialization in their natural state or as derived products in Brazil.

To quantify the Brazilian fruit consumption, the mean intake over the 2 d of 24HR (when available) was estimated. This involved calculating the per capita daily consumption in grams and the energy-adjusted consumption in grams per 1000 kcal. The latter was derived by assessing the grams of fruit consumed per 1000 kcal of an individual’s total energy intake. This method offers insights into both the absolute consumption in grams and the relative proportion consumed relative to total intake, which can vary based on factors such as age, sex, and physical activity level. Moreover, the proportion of consumers was calculated.

Quantifications were conducted at the national level and further stratified by Brazilian macro region (North, Northeast, South, Southeast, and Midwest), sex (female and male), age group (adolescents aged 10–18 y, adults aged 19–59 y, and older adults aged ≥60 y), place of residence (urban and rural), educational level (incomplete elementary school and complete elementary school), income level (<0.5 minimum wage, ≥0.5 and <1 minimum wage, ≥1 and <2 minimum wages, and ≥2 minimum wages), and ethnic group (White, Yellow, Black, Brown, and Indigenous).

For statistical analyses, descriptive assessments of fruit consumption were performed using the fruit quantified in all 3 scenarios. However, the results derived from scenario 3 are presented in greater analytical detail compared with scenarios 1 and 2 because this scenario is considered to provide a more comprehensive and accurate representation of fruit consumption.

Results were expressed through absolute frequencies, means, and 95% confidence intervals (CIs) [31]. Statistical significance was assumed when there was no overlap of the 95% CI of the point estimates. Additionally, complex sampling analyses were undertaken using the sample weights provided in the INA/POF dataset, as estimates are based on the INA 24HR subsample of POF. Therefore, weighted totals represent the population covered by the analytical sample and may differ from the total Brazilian population in the same period.

Efforts were made to adjust for within-individual variability using the method developed by the National Cancer Institute (NCI) with the MIXTRAN and DISTRIB macros in the Statistical Analysis System [32]. The correlated 2-part model (corr) was employed, as the number of nonconsumers exceeded 5% (for total fruit intake and for all 5 fruit subgroups). However, because of the size of the database, the corrected estimate of the SE was not feasible in several cases, as the correlated 2-part NCI model failed to converge during the replication runs used for variance estimation. Without adjustment for intraindividual variability, it is necessary to consider that the presented CI may be larger than expected.

Statistical analyses were performed using R (R Foundation for Statistical Computing) with the *survey* package to account for the complex sampling design.

## Results

A total of 92 fruits were initially identified using the INA/POF 2017–2018 classification system (scenario 1). After harmonizing the dataset and accounting for synonyms (scenario 2), 85 foods were classified as fruits. In addition, no homonyms were identified. At this stage, additional fruits were identified, including olives and various palm fruits, by applying the harmonized food grouping (Table 2). On the other hand, foods originally classified as fruits in the original survey were reclassified into other food groups. These were jackfruit, breadfruit, physalis, sugar cane, and plantains.

**TABLE 2:**
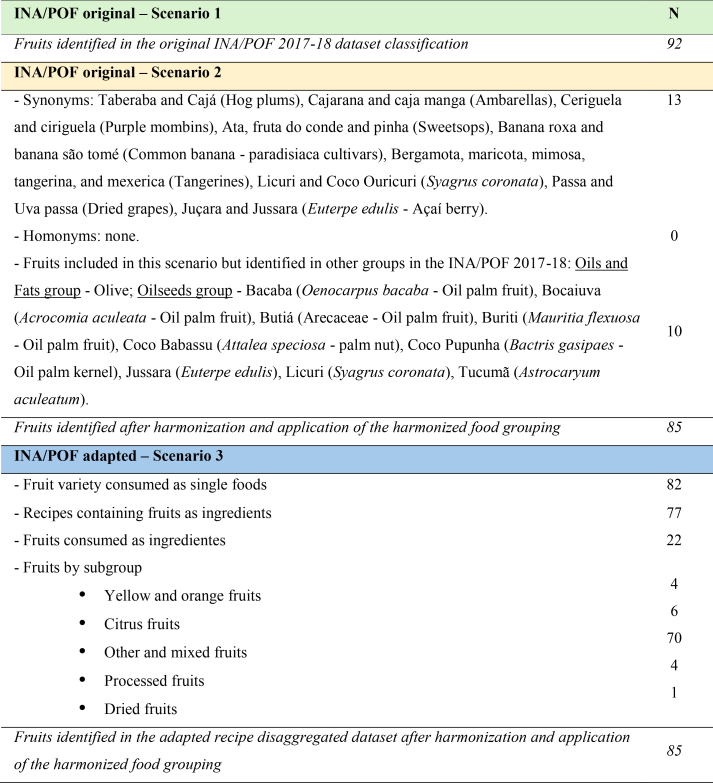
Identification and classification of fruits in the INA/POF 2017–2018 dataset using different scenarios

Abbreviations: INA, Brazilian National Food Consumption Survey; POF, Household Budget Survey.

In scenario 3, which included fruit consumption from recipes, a total of 85 fruits were identified as either consumed individually or as part of recipes (Table 2). Among the 955 different recipes reported in the survey, 8.1% contained fruits. Additionally, 22 of the 85 fruits were used as ingredients, with prunes being the only fruit reported exclusively as an ingredient. In contrast, fruit salad, previously classified as a single fruit item in earlier scenarios, was disaggregated into its individual ingredients. Among the fruits consumed, 70 were fresh, 4 were dried, and 1 was processed (olive, predominantly used in savory recipes). The recipe “açaí with guarana and granola” was not fully disaggregated in the TBCA and was therefore excluded from the assessment because of uncertainty on its fruit content. However, discrete fruits identified only as “açaí” were retained in the dataset.

Of the 92 foods recognized as species from the Brazilian sociobiodiversity, 23 were fruit species reported as consumed in the survey. These native fruits accounted for 27.1% of the total number of fruit items identified in the survey. These fruits include pineapple (*Ananas comosus*), abiu (*Pouteria caimito*), açaí berry (*Euterpe oleracea*), blackberry (*Rubus* spp.), cattley guavas (*Psidium cattleyanum*), mountain soursop (*Annona montana*), platonia (*Platonia insignis*), wild sugar-apple (*Annona squamosa*), cocoa fruit (*Theobroma cacao*), hog plum (*Spondias mombin*), cashew apple (*Anacardium occidentale*), guava (*Psidium guajava*), jenipapo (*Genipa americana*), mangaba (*Hancornia speciosa*), passion fruit (*Passiflora edulis*), nance (*Byrsonima crassifolia*), patawa (*Oenocarpus bataua*), pequi (*Caryocar brasiliense*), surinam cherry (*Eugenia uniflora*), tucumã (*Astrocaryum aculeatum*), umbu (*Spondias tuberosa*), and uxi (*Endopleura uchi*).

**FIGURE 2:**
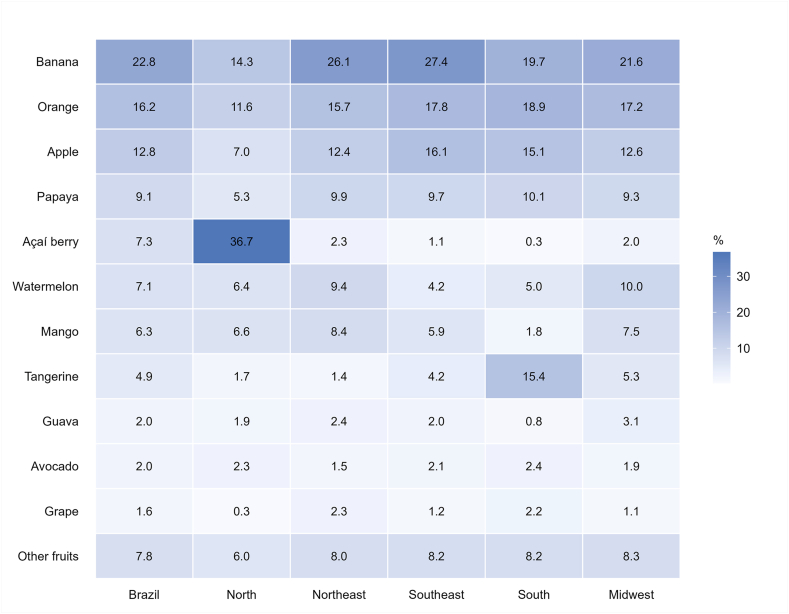
Heatmap of the proportional contribution (%) of the most consumed fruits across Brazilian regions, based on the National Food Consumption Survey (INA/POF) 2017–2018. Scenario 3 (recipes disaggregated at the ingredient level). INA, Brazilian National Food Consumption Survey; POF, Household Budget Survey.

Nationwide, the 5 most commonly consumed fruits in quantities (grams per day) were banana (22.8%), orange (16.2%), apple (12.8%), papaya (9.1%), and açai berry (7.3%). This trend was consistent across most regions, except in the North, where açai (36.7%) consumption exceeded that of banana (14.3%), and in the South, where tangerine consumption was 15.4% in third position. Additional variability across regions was observed for some fruits, such as mango and watermelon, which presented higher contributions in the Northeast and Midwest compared with other regions (Figure 2). Fruit diversity also varied across regions. The Northeast presented the highest number of distinct fruits consumed (*n* = 63), followed by the Southeast (*n* = 59), North (*n* = 57), Midwest (*n* = 55), and South (*n* = 45) (Supplemental Results 1).

Reclassification of the data using the harmonized food grouping (scenario 2) had minimal impact on the overall proportion of fruit consumers compared with the original INA/POF classification (37.0% compared with 37.9%). However, when recipe disaggregation was applied (scenario 3), the proportion of fruit consumers increased substantially to 52.2%. Despite this marked increase in the prevalence of consumption, the mean quantity of fruit consumption changed modestly across scenarios. Mean intake increased from 66.3 g in scenario 1 to 79.8 g in scenario 3. A similar pattern was observed when intake was expressed per 1000 kcal, increasing from 41.3 g/1000 kcal in scenario 1 to 49.7 g/1000 kcal in scenario 3 (Table 3).

**TABLE 3:** Proportion of consumers (%) and mean consumption (in grams and grams per 1000 kcal) of fruit group and subgroups, classified according to different analytical scenarios, in the Brazilian National Food Consumption Survey (INA/POF, 2017–2018)

**Scenario**	**1**	**2**	**3**

**Classification**	**INA/POF**	**Harmonized food grouping**	**Harmonized food grouping**
Recipe disaggregation	No	No	Yes
*Group consumption*	*Proportion of consumers in % (95% confidence interval)*		
Fruit (all)	37.9 (37.0, 38.9)	37.0 (36.0, 37.9)	52.2 (51.2, 53.2)
Yellow and orange fruits	—	7.0 (6.6, 7.5)	16.6 (15.9, 17.3)
Citrus fruits	—	7.9 (7.3, 8.5)	10.4 (9.8, 11.1)
Other and mixed fruits	—	30.5 (29.6, 31.4)	41.3 (40.4, 42.3)
Processed fruits	—	0.2 (0.1, 0.3)	4.1 (3.7, 4.6)
Dried fruits	—	0.2 (0.1, 0.3)	4.4 (4.0, 4.8)
*Group consumption*	*Meanconsumption in grams*		
Fruit (all)	66.3 (63.9, 68.8)	64.5 (62.1, 66.9)	79.8 (77.3, 82.3)
Yellow and orange fruits	—	9.4 (8.7, 10.1)	12.9 (12.1, 13.7)
Citrus fruits	—	13.3 (12.1, 14.4)	14.2 (13.1, 15.4)
Other and mixed fruits	—	39.7 (38.1, 41.4)	52.4 (50.5, 54.2)
Processed fruits	—	0.03 (0.02, 0.05)	0.11 (0.09, 0.13)
Dried fruits	—	0.03 (0.02, 0.05)	0.23 (0.19, 0.26)
*Group consumption*	*Meanconsumption in grams/1000 kcal*		
Fruit (all)	41.3 (39.8, 42.8)	40.2 (38.8, 41.7)	49.7 (48.2, 51.3)
Yellow and orange fruits	—	6.2 (5.7, 6.8)	8.4 (7.8, 8.9)
Citrus fruits	—	8.1 (7.4, 8.7)	8.8 (8.1, 9.4)
Other and mixed fruits	—	24.6 (23.6, 25.6)	32.4 (31.3, 33.5)
Processed fruits	—	0.015 (0.008, 0.021)	0.06 (0.05, 0.07)
Dried fruits	—	0.02 (0.01, 0.03)	0.12 (0.11, 0.14)

Abbreviations: INA, Brazilian National Food Consumption Survey; POF, Household Budget Survey.

At the fruit subgroup level, recipe disaggregation (scenario 3) increased both the proportion of consumers and the mean intake across all fruit categories in comparison with scenario 2 (Table 3). The largest absolute increase in consumer prevalence was observed for “other and mixed fruits” (from 30.5% to 41.3%), followed by yellow and orange fruits (from 7.0% to 16.6%). Citrus fruit consumption also increased, although more modestly (from 7.9% to 10.4%). Likewise, processed and dried fruits, which were previously negligible in scenario 2 (0.2% each), showed a marked increase after recipe disaggregation, reaching 4.1% and 4.4% consumers, respectively. These changes were reflected in intake estimates. The largest absolute increase in both g/d and g/1000 kcal was observed for other and mixed fruits, for which the mean intake increased from 39.7 to 52.4 g/d and from 24.6 to 32.4 g/1000 kcal. Although processed and dried fruits showed the largest relative increase after recipe disaggregation, their contribution to total fruit intake remained small.

In the most detailed approach (scenario 3), the analysis revealed variations in fruit consumption across regions and some sociodemographic groups (Table 4). At the regional level, the highest proportion of fruit consumers was observed in the Northeast (60.8%), whereas the lowest was in the Southeast (47.7%) and the Midwest (47.6%). Mean per capita intake ranged from 69.6 g/d in the Southeast to 93.8 g/d in the North, whereas energy-adjusted intake varied from 45.5 g/1000 kcal/d in the Southeast to 54.7 g/1000 kcal/d in the South.

**Table 4:** Quantification of fruit consumption in Brazil by sociodemographic characteristics, from the National Food Consumption Survey (INA/POF, 2017–2018)^1^

Sociodemographic characteristic	Proportion of fruit consumers		Mean per capita fruit consumption per g/d (95% CI)	Mean per capita fruit consumption per g/1000 kcal/d (95% CI)

*N* (total)	Proportion (95% CI)

Macroregion				
North	7,843,353	53.1 (50.2, 55.9)	93.8 (82.8, 104.8)	51.3 (46.3, 56.3)
Northeast	29,541,748	60.8 (59.3, 62.3)	90.0 (86.1, 93.9)	53.9 (51.7, 56.2)
Southeast	36,632,193	47.7 (45.8, 49.7)	69.6 (56.2, 73.9)	45.5 (42.7, 48.4)
South	13,282,400	51.0 (48.8, 53.1)	86.8 (80.6, 92.9)	54.7 (51.2, 58.3)
Midwest	6,562,204	47.6 (45.1, 50.2)	72.4 (66.0, 78.8)	47.1 (43.0, 51.3)
Sex				
Female	53,554,976	57.1 (55.9, 58.3)	85.9 (83.0, 88.8)	59.5 (57.5, 61.6)
Male	40,306,923	46.8 (45.5, 48.0)	73.1 (70.1, 76.2)	39.1 (37.5, 40.7)
Age group				
Adolescents (10–18 y)	13,773,063	47.6 (45.6, 49.6)	60.7 (56.0, 65.4)	34.6 (32.3, 36.8)
Adults (19–59 y)	60,384,839	50.6 (49.5, 51.7)	77.1 (74.3, 79.8)	46.5 (44.8, 48.2)
Older adults (≥60 y)	19,703,998	62.2 (60.3, 64.0)	107.5 (102.0, 113.0)	75.7 (71.9, 79.6)
Place of residence				
Rural area	14,194,270	54.4 (52.2, 56.6)	85.2 (78.9, 91.4)	49.3 (46.0, 52.6)
Urban area	79,667,629	51.8 (50.7, 52.9)	78.9 (76.1, 81.6)	49.8 (48.1, 51.5)
Educational level				
Incomplete elementary school	36,987,347	49.0 (47.8, 50.3)	72.3 (69.2, 75.5)	46.3 (44.4, 48.2)
Complete elementary school	56,874,553	54.4 (53.1, 55.7)	85.2 (81.9, 88.5)	52.2 (50.1, 54.3)
Income level				
<0.5 MW	10,857,836	43.2 (40.8, 45.7)	59.0 (52.7, 65.3)	35.0 (31.8, 38.1)
≥0.5 and <1 MW	21,598,406	46.1 (44.4, 47.8)	61.0 (57.6, 64.5)	37.9 (35.8, 40.0)
≥1 and <2 MW	33,224,698	53.3 (51.6, 55.0)	78.3 (74.6, 82.1)	49.0 (46.7, 51.4)
≥2 MW	28,140,239	61.8 (59.7, 63.8)	112.5 (106.4, 118.6)	70.9 (67.2, 74.7)
Ethnicity				
White	42,090,707	54.3 (52.8, 55.8)	85.7 (81.8, 89.5)	54.4 (52.0, 56.8)
Yellow	798,310	65.1 (53.2, 75.4)	117.9 (63.2, 172.6)	66.9 (38.8, 95.0)
Black	9,465,792	48.7 (46.0, 51.4)	73.9 (67.8, 80.0)	46.9 (42.4, 51.5)
Brown	41,071,525	50.9 (49.6, 52.1)	75.1 (71.9, 78.4)	46.0 (43.9, 47.6)
Indigenous	365,751	47.3 (37.8, 57.0)	63.3 (38.1, 88.4)	39.5 (22.6, 56.5)

Abbreviation: CI, confidence interval; INA, Brazilian National Food Consumption Survey; MW, minimum wage; POF, Household Budget Survey.

1 Based on scenario 3: after harmonization, recipe disaggregation, and classification according to the harmonized food grouping [21].

Sex differences were also evident, with a higher proportion of female participants consuming fruits compared with male participants (57.1% compared with 46.8%). Female participants also reported higher mean intake (85.9 g/d compared with 73.1 g/d) and higher energy-adjusted consumption (59.5 g/1000 kcal/d compared with 39.1 g/1000 kcal/d), respectively.

Age-related patterns indicated that elderly (age ≥60 y) individuals had the highest prevalence of consumption (62.2%) and the highest mean intake (107.5 g/d; 75.7 g/1000 kcal/d), whereas adolescents presented the lowest values (47.6%; 60.7 g/d; 34.6 g/1000 kcal/d).

Differences by place of residence were less pronounced, with similar proportions of consumers in rural (54.4%) and urban areas (51.8%), as well as comparable fruit consumption levels.

A clear socioeconomic gradient was observed. Both the proportion of consumers and mean intake increased with income, ranging from 43.2%, 59.0 g/d, and 35 g/1000 kcal/d among individuals earning less than half of the minimum wage to 61.8%, 112.5 g/d, and 70.9 g/1000 kcal/d among those earning more than twice the minimum wage. A similar pattern was observed across educational levels, with lower consumption among individuals with less education compared with those with more education (49.0% compared with 54.4%; 72.3 g/d compared with 85.2 g/d; 46.3 g/1000 kcal/d compared with 52.2 g/1000 kcal/d).

Finally, variations were also identified across ethnic groups, with higher consumption among individuals classified as Yellow (65.1%) and White (54.3%), and lower values among Black (48.7%), Brown (50.9%), and Indigenous populations (47.3%). Mean intake was also highest among the Yellow group (117.9 g/d; 66.9 g/1000 kcal/d), although estimates for this group should be interpreted with caution because of wider variability.

## Discussion

The aim of this study was to harmonize and quantify fruit consumption in the Brazilian population using data from the INA/POF 2017–2018 at the ingredient level, while applying the harmonized food grouping. Considering the most detailed dataset (scenario 3), the mean fruit consumption in Brazil was 79.8 g per capita per day (95% CI: 77.3, 82.3) and 49.7 g per 1000 kcal per capita per day (95% CI: 48.2, 51.3), with 52.2% of the population identified as fruit consumers (95% CI: 51.2, 53.2). A total of 85 distinct fruit types and 10 fruits with synonymous names were reported. Ten fruits accounted for 90.6% of all fruits reported consumed, with banana, orange, apple, papaya, and açai berry being the 5 most commonly consumed.

Compared with other LMICs, fruit consumption in Brazil appears broadly comparable. The Latin American Study of Nutrition and Health (ELANS) [33], which collected standardized 24HR on representative samples across 8 Latin American countries (using similar, though not identical fruit classification), reported a mean fruit intake of 75.3 g/d overall, with Brazil at 70.5 g/d, similar to Argentina (74.3 g/d), Colombia (67.9 g/d), and Costa Rica (68.6 g/d), but lower than Ecuador (89.6 g/d), Chile (100.5 g/d), and especially Peru (117.8 g/d). Venezuela presented markedly lower fruit intakes (26.2 g/d). On the other hand, estimates from other studies that collected data using 24HR or food records accessed through the FAO/WHO GIFT platform, and harmonized using the same nutrition-sensitive food grouping as the present study, show higher consumption among similar population groups in countries such as Italy (213 g/d for individuals aged 10–97 y), Peru (167 g/d, 18–59 y), Mexico (126 g/d, 10–98 y), and Romania (175 g/d, 19–92 y), whereas lower values are observed in Saint Kitts and Nevis (57 g/d, 18–65 y), Fiji (46 g/d, 15–49 y), and Bulgaria (36 g/d, 16–95 y) [19].

Global fruit production is highly concentrated, with China (269 million tons), India (114 million tons), and Brazil (43 million tons) ranking as the largest producers in 2023 [34]. Despite being one of the world’s leading fruit producers, Brazil does not exhibit correspondingly high levels of fruit consumption and plays a relatively modest role in the global fruit export market. In 2023, the country exported ∼1.1 million tons of fruit, representing only a small fraction of its total production [35]. This apparent disconnect between production capacity and consumption patterns is reflected in our findings. Although a wide variety of fruits is available in Brazil, fruit consumption was concentrated in a relatively small number of species, with only 27.1% of the fruits identified belonging to recognized sociobiodiversity species. These findings suggest that low fruit consumption in Brazil is accompanied by limited diversity in fruit choices, despite the country’s rich biodiversity and substantial fruit production.

Recipe disaggregation revealed that 8.1% of the survey recipes included fruits, involving 22 different fruit ingredients. This procedure meaningfully increased the proportion of fruit consumers identified in Brazil, although the quantities consumed changed modestly across scenarios, possibly indicating that only small amounts were used as ingredients in most of the preparations. Nevertheless, recipe disaggregation does not always improve the estimation of consumers or quantities consumed. In a recent survey in Saint Kitts and Nevis, using a very similar food grouping, fruit consumption did not change after recipe disaggregation, whereas this was different for several other food groups, such as cereals, vegetables, fish, and meats [17].

This difference in results likely reflects the fact that, in some countries, fruits are predominantly consumed as discrete items rather than incorporated into mixed dishes, which substantially constrains the impact of recipe disaggregation on total intake estimates. As a large and culturally diverse country, regional dietary patterns in Brazil can vary considerably, including the ways fruits are consumed. This heterogeneity may possibly explain why disaggregation contributes to identifying a higher proportion of consumers, even when its effect on total quantities remains modest.

Another explanation relates to the operational definition of recipes within national surveys, because variations in how recipes are defined, recorded, and disaggregated can directly affect the identification of fruit ingredients and, consequently, their contribution to total intake estimates [17]. In the case of INA/POF, a recipe disaggregation methodology originally developed for nutrient estimation and based on different procedures was adapted to quantify food group intakes at the ingredient level [29]. Although this approach enabled a more comprehensive estimation of fruit consumption, it relies on assumptions regarding recipe composition, ingredient proportions, yield factors, and the allocation of ingredients to food groups. As a result, some uncertainty remains regarding the precise contribution of fruit ingredients to total fruit intake estimates.

Furthermore, previous work by Zhang et al. [36], based on the Dutch National Food Consumption Survey, suggests that using standard recipes results in only a minor loss of validity for food group and nutrient estimates. However, the generalizability of these findings remains uncertain. In smaller and more homogeneous settings, such as Saint Kitts and Nevis, this approach may indeed introduce limited bias, whereas in larger and more diverse countries like Brazil, greater variability in culinary practices may result in estimation errors or misclassification of foods incorporated into mixed dishes, such as fruits. With that said, further work is still needed to refine recipe disaggregation procedures and support the production of more harmonized and comparable dietary data across countries.

One of the unique contributions of this study is the identification of 85 distinct fruit types, with a dominant consumption of banana, orange, apple, papaya, and açaí berry. Açaí, in particular, stood out in the North region, where cultural and regional preferences knowingly shape this consumption. The prominence of açaí consumption, particularly in savory dishes (with cassava flour and animal protein) in the North, contrasts with the sweetened, dessert-like preparations popular in other regions, reflecting both cultural diversity and the geographic variation in consumption habits across Brazil. To note, the state of Pará in the North is the largest producer of açaí, accounting for 60% of national production [37].

Sociodemographic factors also play an important role in fruit consumption patterns in Brazil. Female participants and older adults were found to consume more fruits, which aligns with trends reported in the ELANS study in Latin America. Across countries, fruit consumption was consistently higher among female participants than among male participants (e.g., 78.4 compared with 71.9 g/d overall) and increased with age, ranging from 64.4 g/d in adolescents (15–19 y) to 95.7 g/d among older adults (50–65 y) [33]. Interestingly, no significant differences were observed between rural and urban areas in the present study, which contrasts with data from another national survey in Brazil, namely the National Survey of Health (2013) [38], whereby urban populations typically reported a higher proportion of fruit-consuming individuals. This difference may be due to the chosen fruit classification and/or the type of data collected in both surveys (24-h recalls compared with FFQ).

Despite the increase observed after recipe disaggregation, mean fruit intake remained ∼80 g/d, far below the WHO recommendation of ≥400 g/d of fruits and vegetables [39]. There are several factors that could influence the low fruit consumption in Brazil, such as the accessibility of fresh fruits in specific areas, the cost of fruits, time constraints, and dietary habits, among others [40]. Promoting awareness of the health benefits of fruits and implementing public policies that facilitate access to fruits and incentivize their consumption are measures that remain essential to increasing fruit intake in the country.

Because the present analysis focused on whole fruits and fruit ingredients incorporated into foods rather than beverages, a sensitivity analysis was conducted, including fruit juices and fruit-based beverages (results not shown previously). The proportion of fruit consumers remained unchanged (52.2%), whereas mean fruit intake increased only marginally, from 79.8 g/d (95% CI: 77.3 g/d, 82.3 g/d) to 80.7 g/d (95% CI: 78.1 g/d, 83.2 g/d). These findings suggest that the exclusion of fruit juices and fruit-based beverages had a negligible impact on the estimated prevalence of consumers and only a minor effect on mean fruit intake. Therefore, the relatively low fruit consumption observed in Brazil cannot be attributed to the exclusion of beverages from the definition of fruit intake.

The limitations of the study, particularly the high proportion of nonconsumers on both days of dietary recall (over 50%), point to the challenges of accurately capturing habitual fruit consumption. Fruit intake is often episodic rather than daily, and two 24-h recalls may not fully characterize usual intake among infrequent consumers. Although data collection was conducted over a 12-mo period and included different days of the week, residual within-person variation related to temporal factors may not have been completely captured. Furthermore, fruit availability and consumption patterns may vary substantially across regions and seasons in a country as large and diverse as Brazil, and two 24-h dietary recalls may not fully capture these fluctuations. Nevertheless, there is no reason to suggest that seasonality would impact differently the comparison of the 3 scenarios used. The use of complementary methods, such as a food propensity questionnaire for fruit consumption, could improve the robustness of future dietary intake assessments and potentially help address within-person variability, which was not possible in this study [41]. Nevertheless, the mean estimates derived from this study remain informative, as the mean population intake is theoretically unaffected by within-person variability and thus reflects a point estimate of consumption at the population level [42].

Despite the identified limitations, this study offers a comprehensive and nuanced understanding of fruit consumption in Brazil. The recipe disaggregation method allowed us to better capture fruit consumption from culinary preparations, which is often underrepresented in dietary data. Furthermore, the detailed analysis revealed the impact of previously unnoticed food subgroups, such as the consumption of dried fruits used in recipes.

In conclusion, this study provides a more detailed overview of fruit consumption in Brazil and contributes to global efforts to harmonize dietary data. Overall, fruit consumption in Brazil is low, especially in groups with lower income and education levels. In addition, fruit intake is largely restricted to a limited range of fruits, despite the rich diversity of fruits found in different regions of the country. Methodological choices related to applied food groupings and recipe disaggregation seem to substantially influence the identification of fruit consumers and should be carefully considered in dietary assessments. By applying the harmonized food grouping at the ingredient level, this study advances the harmonization of dietary data and provides evidence to support monitoring and policy actions aimed at increasing fruit consumption and improving diet quality in Brazil. Furthermore, the application of harmonized food grouping and ingredient-level recipe disaggregation may also be informative for other countries conducting dietary surveys.

## Author contributions

The authors’ responsibilities were as follows – TRG, GVdS, MCRdA, SPC: designed the research; TRG, GVdS, SPC: conducted the research and wrote the paper; TRG: analyzed data; CCBA, VPdQ, AB, BAH: contributed to data analysis and critically revised the manuscript; SPC: primarily responsible for final content; and all authors: read and approved the final manuscript.

## Data availability

Data described in the manuscript, code book, and analytic code will be made available on request, pending application and approval.

## Declaration of Generative AI and AI-assisted technologies in the writing process

During the preparation of this work, the authors used Perplexity AI (version not specified) to support information retrieval and synthesis. After using this tool, the authors reviewed and edited the content as needed and take full responsibility for the content of the publication.

## Funding

This work was supported by the Coordination for the Improvement of Higher Education Personnel—Brazil—CAPES (Finance Code 001) and the Food and Agriculture Organization of the United Nations (PO number: 363747).

## Conflict of interest

The authors declare no conflict of interest. The views expressed in this publication are those of the authors and do not necessarily reflect the views or policies of the Food and Agriculture Organization of the United Nations.
